# Causal Effects Between Genetically Determined Human Serum Metabolite Levels on the Risk of Idiopathic Pulmonary Fibrosis: A Mendelian Randomization Study

**DOI:** 10.1111/crj.70087

**Published:** 2025-06-13

**Authors:** Yu Shi, Shuang Chen, Zhaokai Zhou, Mengke Huang, Yue Li, Xiaogang Jing

**Affiliations:** ^1^ Department of Respiration The First Affiliated Hospital of Zhengzhou University Zhengzhou Henan China; ^2^ Center of Reproductive Medicine The First Affiliated Hospital of Zhengzhou University Zhengzhou Henan China; ^3^ Department of Urology The First Affiliated Hospital of Zhengzhou University Zhengzhou Henan China

**Keywords:** causality, idiopathic pulmonary fibrosis, Mendelian randomization, serum metabolites

## Abstract

**Background:**

The incidence of idiopathic pulmonary fibrosis (IPF) is increasing every year; however, the potential biological mechanisms have not been completely clarified. The objective of this study is to reveal the etiologic effects of circulating metabolites on IPF.

**Methods:**

This research evaluated the causal relationship between serum metabolites and IPF utilizing two‐sample Mendelian randomization (MR) analysis. IVW served as the main method of analysis; concurrently, MR‐Egger, weighted median, and MR‐PRESSO acted as supplementary analyses. Sensitivity analyses were performed with Cochran's *Q* test, MR‐Egger's intercept test, and leave‐one‐out method of analysis. All statistical analyses were accomplished in R software.

**Results:**

Our results showed that 23 metabolites have a causal connection with IPF. Following sensitivity analysis, 2 robust and 12 potential causal association pairs were identified among the known metabolites. These 14 causal pairs concerned six metabolites.

**Conclusion:**

This study presents a novel perspective on potential mechanisms involved in IPF with important significance for screening, prevention, and treatment.

## Graphical abstract

The objective of this study is to reveal the etiologic effects of circulating metabolites on IPF. This research evaluated the causal relationship between serum metabolites and IPF utilizing two‐sample Mendelian randomization (MR) analysis. This study presents a novel perspective on potential mechanisms involved in IPF with important significance for screening, prevention, and treatment.

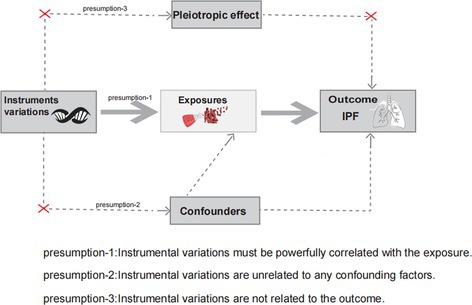

## 1 Introduction

Idiopathic pulmonary fibrosis (IPF) is a chronic, progressive, and irreversible fibrotic lung disease, commonly seen in the elderly, and its etiology has not yet been clarified. The main feature is progressive fibrosis in the lung parenchyma causing deterioration of lung function [1]. IPF is a complex, incurable, and fatal fibrotic lung disease [2]; research has shown that interstitial lung disease is ranked as the 2nd leading cause of death in high‐income countries in Europe and Central Asia [3]. The etiopathology of IPF is characterized by the sustained activation of a high number of fibroblasts and myofibroblasts in lung tissues, characterized by metabolic reprogramming and metabolic dysregulation. In recent years, the morbidity, hospitalization, and mortality rates of IPF have been increasing over time, putting a huge burden on society and becoming a critical public health problem all over the world. Despite some progress in medication, IPF remains a disease without a cure, with median survival being only 2–5 years [1, 4, 5]. The diagnosis of IPF is based on a lung biopsy that indicates typical features and excludes other interstitial lung diseases (ILD) in a multidisciplinary setup [6, 7]. Lung biopsies are invasive, and there are some risky procedures for patients with unsure diagnosis and considered to suffer from IPF [8, 9]. Therefore, it is significant to investigate its prospective biomarkers, diagnose it earlier, and take protective measures.

Recently, genomic technologies including genomics and metabolomics have been extensively practiced to explore the underlying biological mechanisms and treatment strategies of various human disorders. Metabolomics is an emerging high‐throughput technology being used for the testing of minor molecules in biological samples, and it presents new means for better insights into the mechanisms of disease onset [10]. As terminal outputs from upstream genes and proteins, metabolites have the potential to serve as biomarkers representing instantaneous states in individuals. Recent powerful metabolite GWAS have identified metabolite‐disease‐associated loci. These studies have also presented new findings on mechanisms relating these loci to disorders and their associated traits [11]. Additionally, serum metabolites have attracted considerable attention for their role in the normal functioning of the respiratory system. For instance, lipids—a component of serum metabolites—have been shown to be altered in the epithelial lipid metabolism of individuals with asthma [12, 13]. In a guinea pig model, induction with experimental allergic asthma leads to increased ceramide in the airway epithelium [14]. In another study, ceramide levels were found to be augmented in the airway epithelium of patients with cystic fibrosis. Such elevations correlated with cell death, a predisposition to infection, and immune inflammation [15]. The relationship between IPF and serum metabolites remains to be elucidated. Identifying reliable and unique biomarkers would be of significant value for the diagnosis and therapeutic management of IPF [16].

Mendelian randomization (MR) is a research method that has recently gained broad appeal. The fact that randomized controlled trials are so costly, time‐consuming, and difficult to implement has made MR appealing to a wide network of researchers. It functions as an alternative research method depending on two alleles with genetic variants being randomly assigned between generations, mimicking how the random grouping of participants in an RCT study is done [17]. Additionally, genetic irreversibility provides more credible evidence for causal relationships between phenotypes because it removes reverse causation interference [18]. Based on the considerable merit of MR, researchers in the recent decades have utilized openly available GWAS summary statistics to derive causal influences of relevant risk exposures on outcomes. For example, MR methodology has been used to investigate the causal relationships between respiratory conditions and various exposures such as vitamin D levels [19], intestinal microflora [20], circulating C‐reactive proteins [21], smoking [22], and circulating adipokines [23]. In parallel, the scope of GWAS has been gradually expanding [24]. In light of the unclear causal associations between serum metabolites and IPF, further investigation using such approaches is warranted. This study adopted a two‐sample MR approach to comprehensively explore the causal associations between 486 human serum metabolites and IPF risk. Furthermore, metabolites identified as having causal associations are selected as candidate biomarkers to provide deeper insights into the biological processes of IPF. According to our knowledge, this is a comprehensive and systematic MR study evaluating the causal relationship between human blood metabolites and IPF, offering new insights into understanding the role of interactions between genetic and metabolic factors in the etiopathogenesis of human diseases.

## 2 Study Design

**FIGURE 1:**
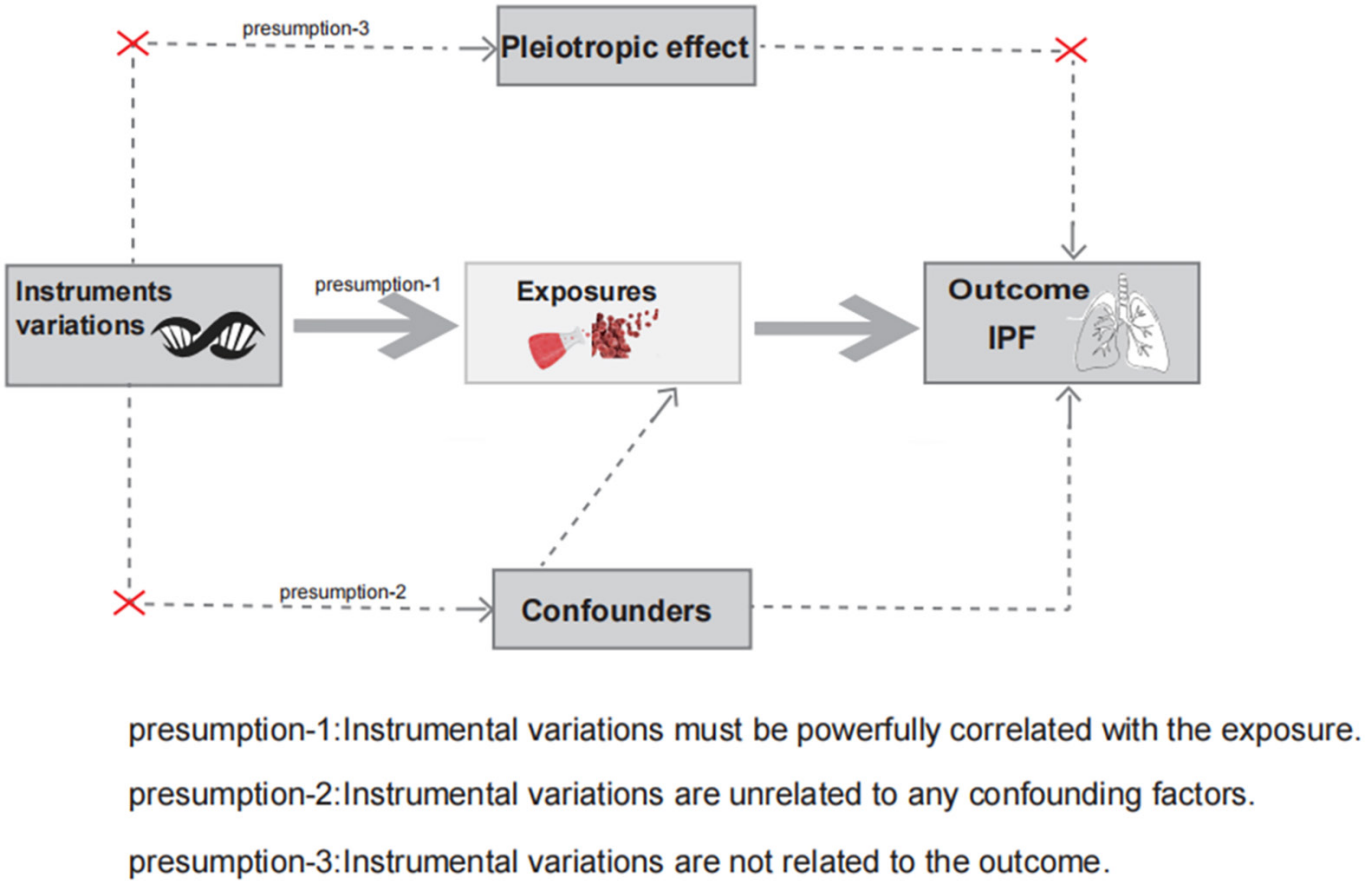
The study design of Mendelian randomization analysis.

The present research was performed based on a strict MR design to comprehensively evaluate 486 serum metabolites correlated with the risk of IPF. A critical and convincing MR study must conform to three presumptions: (1) the presumption of correlation: the associations between genetic instrumental variations and exposure factors must be strongly correlated; (2) the presumption of independence: instrumental variations require independence from confounders, which is labeled as horizontal pleiotropy if it affects the outcome through other factors of risk [25]; and (3) the presumption of exclusion: instrumental variations are not related to the outcome (Figure 1).

### 2.1 GWAS Data for Human Blood Metabolites

We obtained the GWAS data for serum metabolites from (http://metabolomics.helmholtz‐muenchen.de/gwas/), a metabolomics network database. These data were derived by performing GWAS to identify and analyze 2.1 million SNPs containing 309 known metabolites and 177 unknown metabolites. According to the classification system of the Kyoto Encyclopedia of Genes and Genomes (KEGG) database, these 309 known metabolites can be classified into eight different categories: cofactors and vitamins, energy, amino acids, carbohydrates, lipids, nucleotides, peptides, and xenobiotics.

### 2.2 GWAS Data for IPF

The available genetic dataset of IPF for this analysis was obtained from the newly released GWAS meta‐analysis of 1028 cases and 196,986 controls with 16,380,413 SNPs from the Finnish Genome Research Consortium Biobank. The data are publicly accessible on the (https://gwas.mrcieu.ac.uk/) website and therefore do not require additional ethical approval for use.

### 2.3 Selection of Instrumental Variables (IVs)

Having considered multiple aspects, we set screening conditions to ascertain IVs associated with serum metabolites. To follow the first assumption of strong association, we set the significance threshold to 1 × 10^−5^, the linkage disequilibrium (LD) *r* ^2^ to 0.1, and the distance to 500 kb. The *F*‐statistics of the SNPs were then calculated, and SNPs with *F* values of < 10 were excluded, followed by the removal of SNPs correlated with IPF (*p* < 1 × 10^−5^) to meet the third assumption.

### 2.4 Primary Analysis

To explore causal relationships between serum metabolites and IPF, we conducted a two‐sample MR analysis, primarily assessed utilizing the most superior inverse‐variance weighted (IVW) method, which is based on the premise that each genetic variation satisfies the IV assumptions. Potential metabolites were identified by a significance threshold of 0.05, whereas strong metabolites were identified by a Bonferroni‐corrected *p* value of 1.03 × 10^−4^ (0.05/486). In addition, MR estimates of potential metabolites (IVW, *p* < 0.05) were evaluated in three other MR models, i.e., MR‐Egger, weighted median (WM), and MR‐PRESSO. If the estimates of these MR models for a metabolite were similar and satisfied the significance threshold of 0.05 in at least three of the models, including the IVW model, then the metabolite was declared a candidate metabolite.

Afterward, we performed sensitivity analyses to ensure the robustness of the results; we used Cochran's *Q* statistic–IVW and MR‐Egger to determine whether heterogeneity existed in the MR analyses. *p* > 0.05 indicates that there is no heterogeneity, and the MR‐PRESSO global test and the MR‐Egger intercept test were used to evaluate horizontal pleiotropy of genetic variations, followed by leave‐one‐out to ascertain whether a single SNP affects the inference of causality. Additionally, to avoid reverse MR, we ran the Steiger test.

## 3 Results

Out of these 486 serum metabolites we probed, the number of selected IVs ranged from 3 to 481. All SNPs associated with the metabolites showed *F*‐statistics greater than 10.

### 3.1 Primary Analysis

**FIGURE 2:**
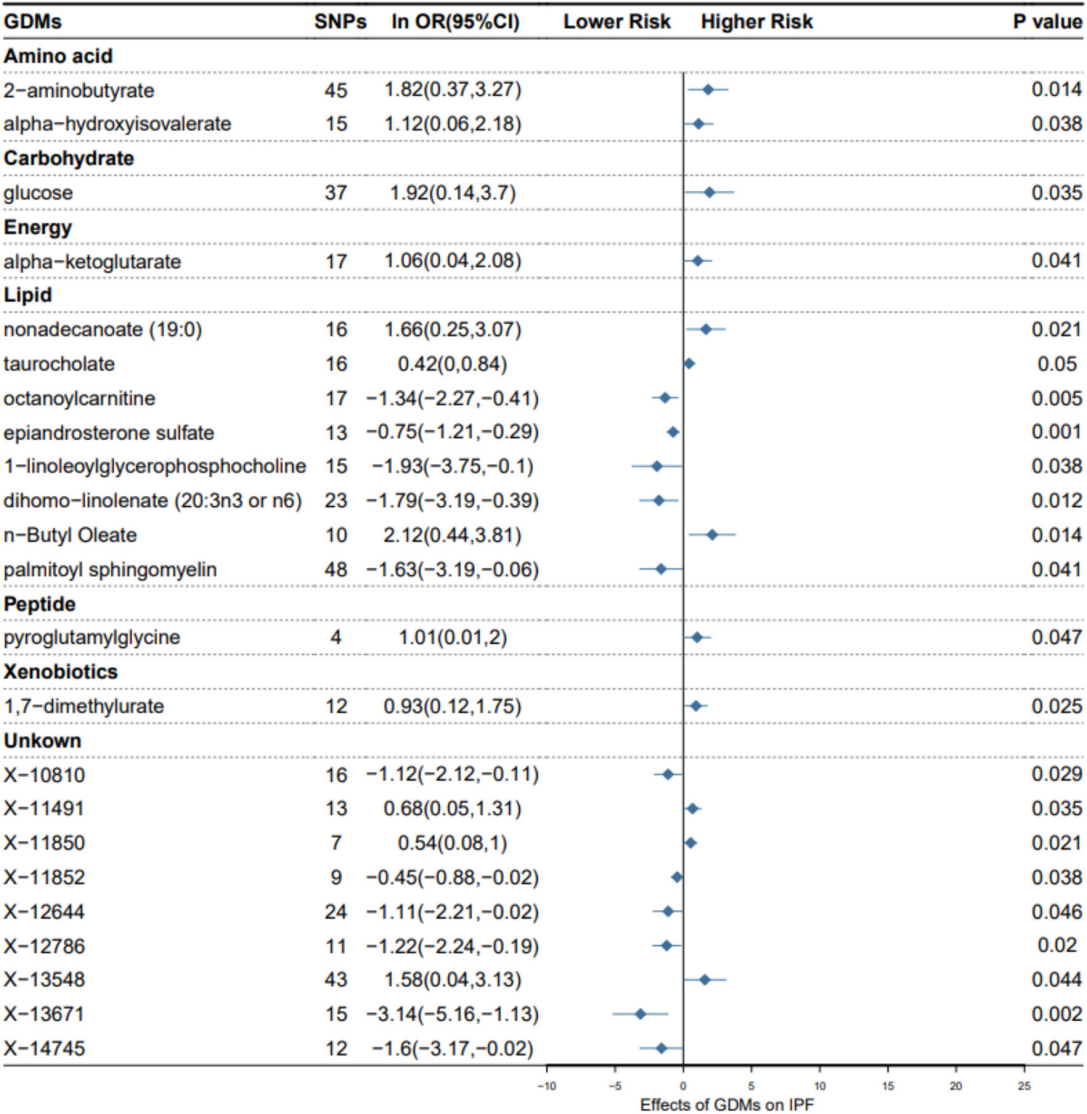
Mendelian randomization associations between serum metabolites and IPF based on IVW.

IVW is the primary MR analysis method. Through this method, our study identified 23 serum metabolites significantly associated with IPF, among which nine chemical compositions have not been distinguished, and the rest 14 belonged to categories: amino acids, carbohydrates, energy, lipids, peptides, and xenobiotics. Specifically, serum metabolites categorized as amino acids, carbohydrates, energy, peptides, and xenobiotics showed positive causal effects on IPF by IVW analysis: alpha–hydroxyisovalerate (odds ratio (OR) = 3.063, 95% confidence interval (95% CI) 1.066–8.804, *p* = 0.038), alpha–ketoglutarate (OR = 2.888, 95% CI 1.044–7.987, *p* = 0.041), pyroglutamylglycine (OR = 2.737, 95% CI 1.013–7.397, *p* = 0.047), 1,7‐dimethylurate (OR = 2.545, 95% CI 1.127–5.745, *p* = 0.025); lipid serum metabolites present different trends and partially show a positive causal effect on IPF: taurocholate (OR = 1.522, 95% CI 1.000–2.314, *p* = 0.050). Other parts show negative causal effects: octanoylcarnitine (OR = 0.261, 95% CI 0.103–0.662, *p* = 0.005), epiandrosterone sulfate (OR = 0.472, 95% CI 0.298–0.745, *p* = 0.001), 1–linoleoylglycerophosphocholine (OR = 0.146, 95% CI 0.024–0.901, *p* = 0.038), dihomo–linolenate (20:3n3 or n6) (OR = 0.167, 95% CI 0.041–0.677, *p* = 0.012), and palmitoyl sphingomyelin (OR = 0.196, 95% CI 0.041–0.938, *p* = 0.041) (Figure 2).

### 3.2 Sensitive Analysis

**FIGURE 3:**
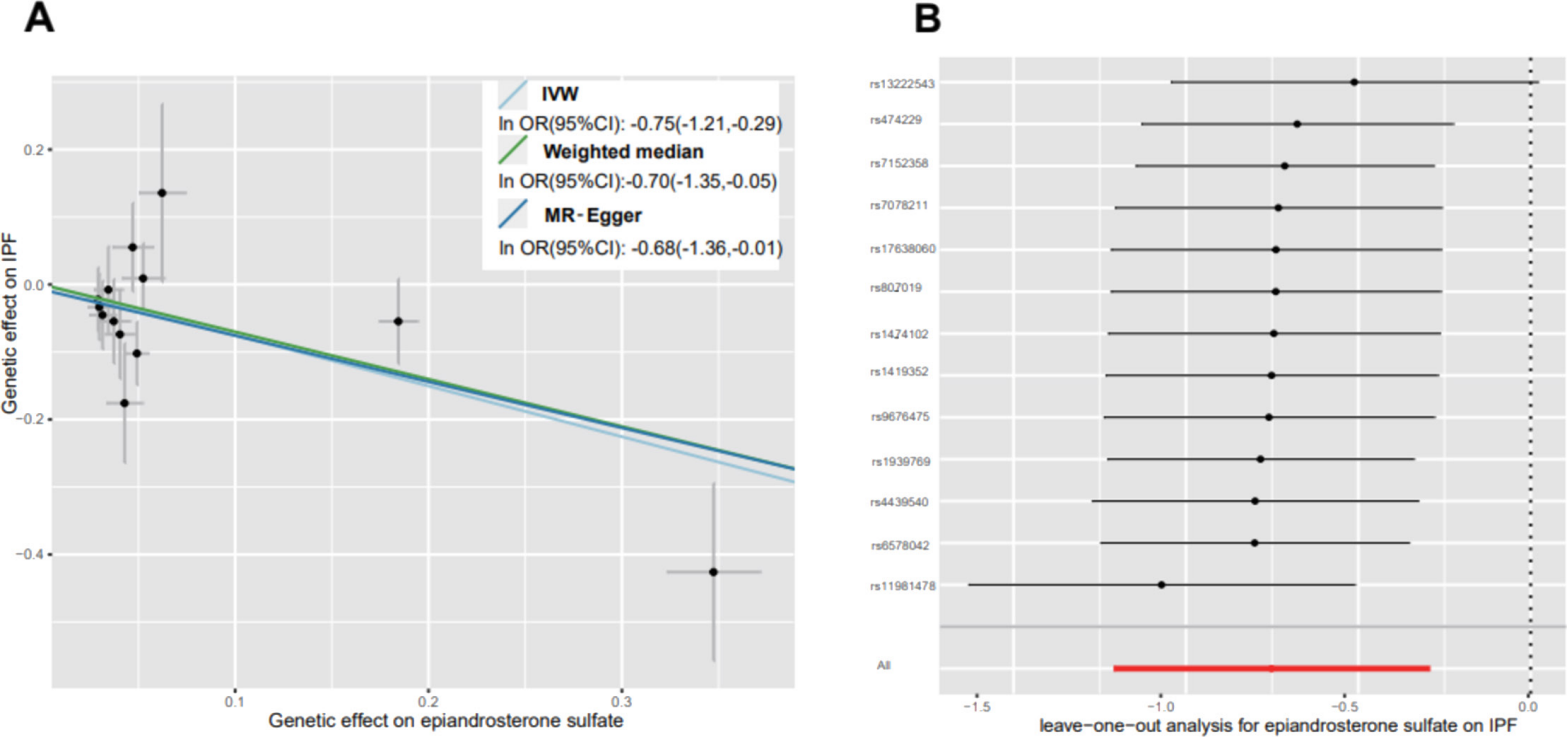
Sensitivity analysis (*p* < 0.05) of genetic associations of epiandrosterone sulfate on IPF. (A) Scatter plot of potential effects of single‐nucleotide polymorphisms (SNPs) on epiandrosterone sulfate vs. IPF, with the slope of each line corresponding to the estimated MR effect per method. SNPs showing negative signals with epiandrosterone sulfate are plotted after orientation to the exposure‐increasing allele. (B) Leave‐one‐out analysis for the impact of individual SNPs on the association between epiandrosterone sulfate and IPF risk. By leaving out exactly one SNP, it depicts how each SNP influences the overall estimate.

Sensitivity analyses were conducted as shown in Table 1. Generally, causality was robust when statistical significance (*p* < 0.05) was observed for two additional MR tests (most often the WM and MR‐PRESSO tests) and the IVW, MR‐Egger, and WM estimates were in the same direction, among which, epiandrosterone sulfate and *n*‐butyl oleate conformed to this condition, had a robust causal relationship with IPF, and could be labeled as candidate metabolites. As an example, epiandrosterone sulfate, which has the most significant IVW results, is presented (Figure 3A).

**TABLE 1:** Sensitivity analysis of causal associations between IVW‐identified metabolites and IPF.

GDMs	MR‐Egger		Weighted median		MR‐PRESSO	
	lnOR (95% CI)	*p*	lnOR (95% CI)	*p*	lnOR (95% CI)	*p*
Amino acid						
2‐Aminobutyrate	2.73 (−1.70, 7.16)	0.2340	1.39 (−0.76, 3.53)	0.2105	1.82 (0.62, 3.02)	**0.0048**
Alpha‐hydroxyisovalerate	−0.69 (−3.70, 2.32)	0.6595	0.42 (−1.13, 1.97)	0.5968	1.12 (0.08, 2.16)	0.0538
Carbohydrate						
Glucose	1.98 (−1.74, 5.70)	0.3034	1.35 (−1.30, 4.00)	0.3351	1.92 (0.31, 3.53)	**0.0253**
Energy						
Alpha‐ketoglutarate	1.80 (−0.25, 3.86)	0.1065	1.46 (−0.07, 3.00)	0.0700	1.06 (0.14, 1.98)	**0.0378**
Lipid						
Nonadecanoate (19:0)	2.38 (−0.28, 5.04)	0.1019	1.47 (−0.49, 3.43)	0.1435	1.65 (0.48, 2.84)	**0.0147**
Taurocholate	0.30 (−0.41, 1.02)	0.4192	0.56 (−0.12, 1.24)	0.0904	0.42 (0.01, 0.83)	0.0629
Octanoylcarnitine	−0.70 (−2.39, 0.99)	0.4315	−0.76 (−2.02, 0.50)	0.2518	−1.34 (−2.27, −0.41)	**0.0121**
Epiandrosterone sulfate	−0.68 (−1.36, −0.01)	0.0738	−0.70 (−1.35, −0.05)	**0.0323**	−0.75 (−1.21, −0.29)	**0.0073**
1‐Linoleoylglycerophosphocholine	−3.47 (−8.54, 1.60)	0.2030	−0.85 (−3.45, 1.75)	0.5071	−1.93 (−3.40, −0.45)	**0.0227**
Dihomo‐linolenate (20:3n3 or n6)	−0.68 (−4.90, 3.55)	0.7571	−1.44 (−3.49, 0.61)	0.1679	−1.79 (−3.19, −0.39)	**0.0201**
*n*‐Butyl oleate	4.10 (−0.31, 8.5)	0.1058	3.36 (0.97, 5.75)	**0.0049**	2.12 (0.49, 3.76)	**0.0315**
Palmitoyl sphingomyelin	3.62 (−2.59, 9.82)	0.2592	−1.19 (−3.42, 1.04)	0.3100	−1.63 (−3.05, −0.21)	**0.0291**
Peptide						
Pyroglutamylglycine	0.98 (−2.11, 4.07)	0.5980	1.05 (−0.23, 2.23)	0.1043	1.00 (0.28, 1.73)	0.0727
Xenobiotics						
1, 7‐Dimethylurate	0.90 (−0.56, 2.36)	0.2557	1.03 (−0.11, 2.17)	0.1039	0.93 (0.37, 1.49)	**0.0075**
Unknown						
X‐10 810	−0.81 (−2.26, 0.64)	0.2898	−1.13 (−2.53, 0.26)	0.1282	−1.12 (−1.86, −0.36)	**0.0110**
X‐11 491	0.56 (−0.71, 1.84)	0.4049	0.40 (−0.51, 1.32)	0.3576	0.68 (0.05, 1.31)	0.0569
X‐11 850	0.70 (−0.08, 1.48)	0.1397	0.50 (−0.09, 1.09)	0.1016	0.54 (0.18, 0.89)	**0.0246**
X‐11 852	−0.91 (−1.57, −0.26)	**0.0296**	−0.38 (−0.98, 0.22)	0.2118	−0.45 (−0.86, −0.04)	0.0632
X‐12 644	−2.17 (−4.54, 0.21)	0.0874	−1.84 (−3.37, −0.32)	**0.0173**	−1.11 (−2.21, −0.02)	0.0584
X‐12 786	−0.98 (−2.83, 0.87)	0.3254	−1.02 (−2.45, 0.41)	0.1660	−1.22 (−2.00, −0.43)	**0.0129**
X‐13 548	3.13 (−1.31, 7.56)	0.1746	1.15 (−1.12, 3.42)	0.3099	1.58 (0.14, 3.03)	**0.0373**
X‐13 671	−0.40 (−8.44, 7.64)	0.9242	−2.04 (−5.02, 0.93)	0.1420	−3.14 (−4.94, −1.34)	**0.0042**
X‐14 745	−4.19 (−13.86, 5.48)	0.4159	−1.25 (3.43, 0.93)	0.2522	−1.60 (−3.17, −0.02)	0.0720

*Note:* Values in bold represent statistically significant data with p 〈 0.05.

Despite associations of the remaining 12 known metabolites with IPF not presenting a more robust causal association in other MR tests, they can still be considered potential causal associations because of the significance observed in IVW methods. We conducted further the Cochran's *Q* test and the MR‐Egger intercept and MR‐PRESSO global test to explore whether there was heterogeneity and horizontal pleiotropy in the results; Cochran's *Q* test showed no heterogeneity (*Q*_pval > 0.05) (Table 2), and the MR‐Egger intercept and MR‐PRESSO global test results also did not show the presence of horizontal pleiotropy (*p*_intercept_ > 0.05 and *p*_global test_ > 0.05) (Table 3). Afterward, we also performed a leave‐one‐out test, with no significant SNP effect on causality, and the metabolite epiandrosterone sulfate is shown in Figure 3B.

**TABLE 2:** Results of heterogeneity by Cochran's *Q* test.

GDMs	Cochran's *Q* test via IVW			Cochran's *Q* test via MR‐Egger		
	*Q*	*Q*_df	*Q*_pval	*Q*	*Q*_df	*Q*_pval
Amino acid						
2‐Aminobutyrate	30.112	44	0.945	29.930	43	0.935
Alpha‐hydroxyisovalerate	13.648	14	0.476	12.058	13	0.523
Carbohydrate						
Glucose	29.455	36	0.772	29.453	35	0.733
Energy						
Alpha‐ketoglutarate	13.022	16	0.671	12.361	15	0.651
Lipid						
Nonadecanoate (19:0)	10.459	15	0.790	10.070	14	0.757
Taurocholate	14.335	15	0.500	14.176	14	0.437
Octanoylcarnitine	17.460	16	0.356	16.569	15	0.345
Epiandrosterone sulfate	13.581	12	0.328	13.488	11	0.263
1‐Linoleoylglycerophosphocholine	9.174	14	0.820	8.765	13	0.790
Dihomo‐linolenate (20:3n3 or n6)	22.865	22	0.409	22.541	21	0.369
*n*‐Butyl oleate	8.480	9	0.487	7.577	8	0.476
Palmitoyl sphingomyelin	35.688	46	0.864	38.619	47	0.803
Peptide						
Pyroglutamylglycine	1.599	3	0.660	1.599	2	0.450
Xenobiotics						
1,7‐Dimethylurate	5.197	11	0.921	5.194	10	0.878
Unknown						
X‐10 810	8.479	15	0.903	8.149	14	0.881
X‐11 491	12.463	12	0.409	12.414	11	0.333
X‐11 850	3.603	6	0.730	3.359	5	0.645
X‐11 852	7.386	8	0.496	4.107	7	0.767
X‐12 644	25.789	23	0.311	24.709	22	0.311
X‐12 786	5.887	10	0.825	5.797	9	0.760
X‐13 548	36.737	42	0.701	36.209	41	0.683
X‐13 671	11.241	14	0.667	10.763	13	0.631
X‐14 745	11.723	11	0.385	11.400	10	0.327

**TABLE 3:** Results of horizontal pleiotropy by the MR‐Egger intercept test and MR‐PRESSO global test.

GDMs	MR‐Egger intercept test			MR‐PRESSO global test	
	Intercept	SE	*p*	Rssobs	*p*
Amino acid					
2‐Aminobutyrate	−0.010	0.023	0.672	31.444	0.939
Alpha‐hydroxyisovalerate	0.056	0.044	0.230	16.554	0.444
Carbohydrate					
Glucose	−0.001	0.020	0.970	30.999	0.799
Energy					
Alpha‐ketoglutarate	−0.022	0.027	0.429	14.083	0.731
Lipid					
Nonadecanoate (19:0)	−0.018	0.030	0.543	11.769	0.798
Taurocholate	0.010	0.025	0.699	16.715	0.528
Octanoylcarnitine	−0.022	0.025	0.383	23.187	0.313
Epiandrosterone sulfate	−0.007	0.027	0.788	19.490	0.347
1‐Linoleoylglycerophosphocholine	0.029	0.045	0.534	11.035	0.778
Dihomo‐linolenate (20:3n3 or n6)	−0.021	0.038	0.588	24.927	0.440
*n*‐Butyl oleate	−0.044	0.046	0.370	10.527	0.501
Palmitoyl sphingomyelin	−0.051	0.030	0.094	40.314	0.822
Peptide					
Pyroglutamylglycine	0.002	0.092	0.987	3.064	0.682
Xenobiotics					
1,7‐Dimethylurate	0.002	0.033	0.956	6.083	0.927
Unknown					
X‐10 810	−0.013	0.022	0.575	8.780	0.935
X‐11 491	0.006	0.030	0.840	15.115	0.421
X‐11 850	−0.018	0.037	0.643	4.491	0.794
X‐11 852	0.060	0.033	0.113	12.259	0.410
X‐12 644	0.024	0.025	0.337	28.031	0.340
X‐12 786	−0.009	0.030	0.771	6.748	0.859
X‐13 548	−0.017	0.023	0.471	38.748	0.676
X‐13 671	−0.037	0.054	0.502	12.765	0.712
X‐14 745	0.058	0.109	0.606	13.737	0.426

To check whether our directions were correct, we performed a Steiger test, which showed that no directional problem was observed in all (Table 4).

**TABLE 4:** Estimation of the Steiger direction from serum metabolites to IPF.

GDMs	SNP_r2. exposure	SNP_r2. outcome	Direction	Steiger *p*
Amino acid				
2‐Aminobutyrate	0.1466	1.83e‐04	TRUE	1.81e‐235
Alpha‐hydroxyisovalerate	0.0910	9.07e‐05	TRUE	1.05e‐119
Carbohydrate				
Glucose	0.1505	1.71e‐04	TRUE	5.02e‐224
Energy				
Alpha‐ketoglutarate	0.0883	8.68e‐05	TRUE	3.03e‐101
Lipid				
Nonadecanoate (19:0)	0.0829	7.96e‐05	TRUE	1.41e‐105
Taurocholate	0.2139	9.19e‐05	TRUE	1.55e‐147
Octanoylcarnitine	0.1089	1.32e‐04	TRUE	4.35e‐171
Epiandrosterone sulfate	0.1233	1.28e‐04	TRUE	1.18e‐181
1‐Linoleoylglycerophosphocholine	0.0725	6.80e‐05	TRUE	3.50e‐96
Dihomo‐linolenate (20:3n3 or n6)	0.0979	1.48e‐04	TRUE	4.00e‐142
*n*‐Butyl oleate	0.0505	7.36e‐05	TRUE	4.93e‐45
Palmitoyl sphingomyelin	0.1503	2.16e‐04	TRUE	8.10e‐240
Peptide				
Pyroglutamylglycine	0.0793	2.80e‐05	TRUE	3.25e‐22
Xenobiotics				
1,7‐Dimethylurate	0.0951	5.18e‐05	TRUE	3.30e‐95
Unknown				
X‐10 810	0.0653	6.68e‐05	TRUE	5.11e‐88
X‐11 491	0.0956	8.62e‐05	TRUE	5.02e‐113
X‐11 850	0.0460	4.52e‐05	TRUE	3.24e‐41
X‐11 852	0.0995	5.90e‐05	TRUE	6.42e‐53
X‐12 644	0.0981	1.53e‐04	TRUE	1.14e‐139
X‐12 786	0.0618	5.69e‐05	TRUE	5.35e‐70
X‐13 548	0.1940	2.06e‐04	TRUE	3.93e‐244
X‐13 671	0.0512	1.04e‐04	TRUE	5.93e‐72
X‐14 745	0.0491	8.05e‐05	TRUE	2.44e‐54

## 4 Discussion

In this study, we conducted a two‐sample MR analysis to explore the causal association of 486 serum metabolites with IPF. We utilized large‐scale IPF GWAS summary data, employing genetic variation as IVs and analyzing them using the IVW method. Ultimately, we identified 23 serum metabolites causally associated with IPF, of which 14 are known metabolites. Through sensitivity analysis, we identified two metabolites with more robust causal relationships among the 14 metabolites. We designated these two metabolites as candidate metabolites. The results showed that higher levels of *n*‐butyl oleate are causally associated with an increased risk of IPF and that higher levels of epiandrosterone sulfate play a protective role in the development of IPF.

Some studies suggest that changes in the metabolism of certain nonessential amino acids drive a profibrotic cellular phenotype in IPF [26], characterized by progressive fibrosis in IPF mainly due to the activation of fibroblasts, a process that is regulated by a variety of lipid metabolites [27, 28, 29]. With the swift development of metabolomics, the pathophysiologic mechanisms of the disease are being better clarified.

It has been shown that alpha‐hydroxyisovalerate is related to the severity of capillary bronchitis [30], along with 2‐aminobutyric acid, a serum metabolite belonging to the same group of amino acids, increasing the risk of developing IPF, yet there are fewer studies on their relevance to IPF, with the detailed mechanisms still to be clarified and explored in depth. Glucose exerts an indispensable role in cellular function, as it turns out that levels of myofibroblast glycolytic metabolism are increased in IPF, and elevated glycolysis stabilizes hypoxia‐inducible factor 1 alpha, an essential ingredient for myofibroblast differentiation [31], which is in agreement with our results: higher levels of glucose are a hazardous condition for IPF. Incidentally, elevated levels of alpha‐ketoglutarate (α‐ketoglutarate) also show an increased risk of IPF, in accordance with existing studies. α‐Ketoglutarate is a critical intermediate in the tricarboxylic acid cycle (TCA), as previously suggested by studies showing that, in lung fibroblasts, transforming growth factor β1 activates glutamine breakdown to convert glutamine to α‐ketoglutarate as a way to increase the metabolites of the TCA, hence facilitating the metabolic reprogramming processes required for the differentiation of myofibroblasts [32], increasing vulnerability to IPF. Fatty acids are widespread in life forms and act in nature. Epiandrosterone sulfate is a steroid hormone found to have the most significant IVW results in our study and may be concerned with reducing IPF risk. Previous studies have linked it to various diseases, such as lowering Alzheimer's disease risk [33], raising osteoporosis risk [34], and polycystic ovary syndrome [35], even though detailed mechanisms remain to be clarified, and it may be a potential biomarker to be used in the future. Available studies related to other lipid metabolites indicate taurocholate has potential in treating inflammatory bowel disease [36]; octanoylcarnitine and other medium‐chain acylcarnitines reduced in IPF [37], as indicated by our results, may offer a protective effect on IPF; nonadecanoate (19:0) may be linked to lowered colorectal cancer risk [38], with more studies related to the digestive system; 1‐linoleoylglycerophosphocholine has been found to be associated with a variety of disorders and could be a potential biomarker for patients with liver disease [39], related to Hunner‐type interstitial cystitis [40] and osteoporosis [41]; 1‐linoleoylglycerophosphocholine has diagnostic value in identifying the degree of ischemia–reperfusion injury based on cardiac biomarkers [42]; dihomo‐linolenate increases cavernous stroke risk [43], which is useful in blood pressure management [44]; *n*‐butyl oleate is functionally related to butan‐1‐ol and oleic acid, respectively, with studies showing strong anticancer activity in *n*‐butyl derivatives [45] and great potential in cancer treatment; palmoyl sphingomyelin is a common form of sphingomyelin that is a hazardous factor for cardiovascular diseases [46]. Pyroglutamylglycine, belonging to the peptide family, is functionally related to glycine and 5‐oxo‐L‐proline, finding a probable association with pancreatic ductal adenocarcinoma [47]. Besides, 1,7‐dimethylurate was found as caffeine metabolite in a number of studies [48, 49], relatively few of them related to IPF, and the detailed mechanisms need to be further explored, with promise to be potential biomarkers for IPF in the future.

Nonetheless, there are some limitations in this study. First, all data were drawn from European populations; though it avoids population heterogeneity, it restricts the application of the results to other populations. Second, nine of the potential biomarkers of IPF identified in this study are metabolites of unknown biological composition and function, limiting interpretations to some extent. Third, although we obtained 14 metabolites causally related to IPF, numerous detailed mechanisms remain to be explored in more in‐depth studies.

Overall, this study systematically evaluated 486 potential causal relationships and provided evidence of a causal relationship between 14 known metabolites and IPF for the first time. Our findings suggest that these metabolites can be regarded as useful biomarkers for IPF screening in clinical practice as well as presenting a reference direction for mechanistic explorations in future cohort and experimental research.

## Author Contributions

Y.S. and X.G.J. contributed to the study design and data analysis. Y.S. wrote and edited the manuscript. Y.S., S.C., Z.K.Z., M.K.H., and Y.L. contributed to project oversight and manuscript revisiting. All authors read and approved the final version.

## Consent

All authors read and approved the final version of the manuscript for publication.

## Conflicts of Interest

The authors declare no conflicts of interest.

## Data Availability

Data are available at http://metabolomics.helmholtz‐muenchen.de/gwas/ and https://gwas.mrcieu.ac.uk/.
